# Research gaps and future needs for allergen prediction in food safety

**DOI:** 10.3389/falgy.2024.1297547

**Published:** 2024-02-19

**Authors:** A. Fernandez, E. Danisman, M. Taheri Boroujerdi, S. Kazemi, F. J. Moreno, M. M. Epstein

**Affiliations:** ^1^ European Food Safety Authority (EFSA), Parma, Italy; ^2^ Experimental Allergy Laboratory, Department of Dermatology, Medical University of Vienna, Vienna, Austria; ^3^ Instituto de Investigación en Ciencias de la Alimentación (CIAL), CSIC-UAM, CEI (UAM+CSIC), Madrid, Spain

**Keywords:** sensitisation, elicitation, bioinformatics, predictive, risk assessment, allergy, protein safety, food allergy

## Abstract

The allergenicity and protein risk assessments in food safety are facing new challenges. Demands for healthier and more sustainable food systems have led to significant advances in biotechnology, the development of more complex foods, and the search for alternative protein sources. All this has increased the pressure on the safety assessment prediction approaches anchored into requirements defined in the late 90's. In 2022, the EFSA's Panel on Genetically Modified Organisms published a scientific opinion focusing on the developments needed for allergenicity and protein safety assessments of new products derived from biotechnology. Here, we further elaborate on the main elements described in this scientific opinion and prioritize those development needs requiring critical attention. The starting point of any new recommendation would require a focus on clinical relevance and the development of a fit-for-purpose database targeted for specific risk assessment goals. Furthermore, it is imperative to review and clarify the main purpose of the allergenicity risk assessment. An internationally agreed consensus on the overall purpose of allergenicity risk assessment will accelerate the development of fit-for-purpose methodologies, where the role of exposure should be better clarified. Considering the experience gained over the last 25 years and recent scientific developments in the fields of biotechnology, allergy, and risk assessment, it is time to revise and improve the allergenicity safety assessment to ensure the reliability of allergenicity assessments for food of the future.

## 1 Introduction

More than 400 genetically modified organisms (GMOs) have been approved worldwide (1) (Supplementary Material). Since the early 2000s, over 100 GMOs have been approved in the European Union (EU) (2, 3). To date, EFSA's allergenicity risk assessment for approved GMOs has not identified any hazards. However, the scientific community is facing new challenges, starting with the population's demands for healthier and more sustainable systems (4–7), leading to significant advances in biotechnology and the development of more complex foods, like products with multiple events containing a high number of new proteins, that, in some cases, are also difficult to test, e.g., membrane-bound proteins, transcription factors; and in a broader context, the assessment of proteins in a new whole food, such as insects. Consequently, the prediction of potential adverse allergic reactions to novel proteins (allergenicity) becomes more difficult.

**Figure 1:**
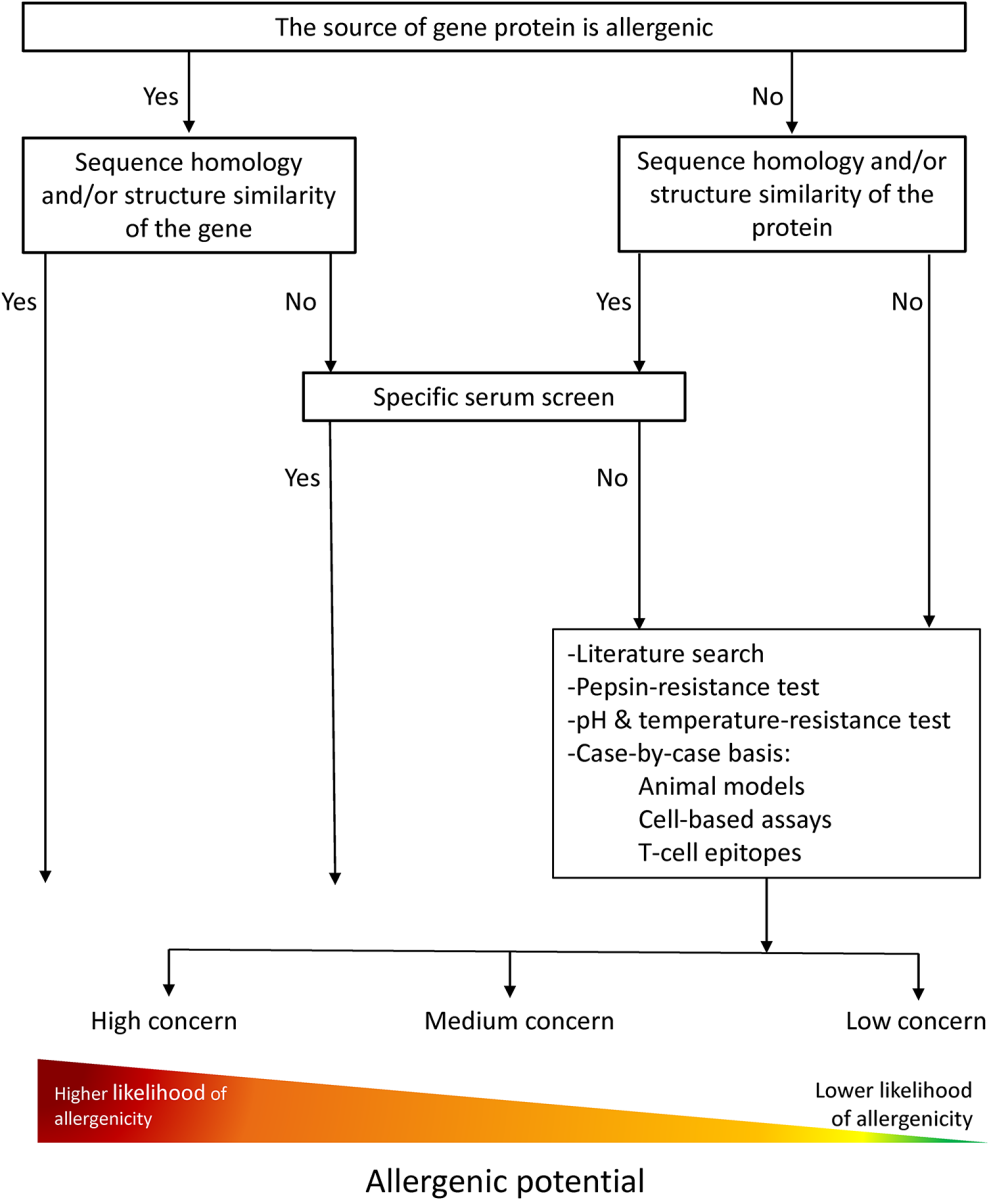
Allergenicity risk assessment current flow chart modified from FAO/WHO (2001) and Davies H. (2005). A weight-of-evidence approach is followed where information of different nature, e.g. *in silico*, *in vitro*, *in vivo*, is considered in the overall assessment to conclude on the allergenic potential of novel proteins.

The current strategies for the allergenicity and safety assessments of new/novel proteins are based on principles adapted from the chemical risk assessment and guidelines of Codex Alimentarius for the safety assessment of foods derived from “modern” biotechnology from 2003 (Figure 1). The assessment is performed for newly expressed proteins in GMOs as well as for whole novel foods. The weight-of-evidence approach is the most robust strategy used for all products, as no single piece of information or experimental method provides sufficient evidence for assessing allergenicity.

In 2022, EFSA published a scientific opinion focusing on the development needs for the allergenicity and protein safety assessment of food and feed products derived from biotechnology (8). A series of short-term and long-term recommendations were provided. These would include the need to: (i) update *in silico* tools that are linked to more targeted databases, (ii) better integrate and standardise test materials and *in vitro/in vivo* assays, (iii) better clarity on the use of the weight-of-evidence approach for protein safety and the role of expert judgment, and (iv) (re)define the allergenicity safety objectives.

Here, we follow-up the EFSA scientific opinion and prioritise the main research gaps and future needs for *in silico*, *in vitro* and *in vivo* allergenicity assessment tools, and other elements, such as dietary exposure, that needs urgent development. It is timely and necessary to revise and improve the allergenicity safety assessment.

## 2 Allergenicity prediction in risk assessment—current state and development needs

### 2.1 *In silico* analysis

Primary amino acid sequence similarity searches against an allergen database are still the current practice for the *in silico* assessment of a novel protein and allergenicity prediction (Figure 1). A threshold value of >35% amino acid identity over at least 80 amino acids was established by a joint FAO/WHO expert consultation in 2001 (9) and embedded in Codex Alimentarius (10). This strategy is considered highly conservative and demanding when hits above the threshold are identified. Furthermore, these *in silico* tools used in the allergenicity assessment inform about the potential capacity of a protein to cross-react with a known allergen (e.g., cross-react and elicit a response in a previously sensitized individual), but they do not provide information on the capacity of proteins for *de novo* sensitization.

Advanced bioinformatic tools different from those defined by Codex (10), the classical FASTA algorithm, are for example similarity searches of 3D protein structure (11, 12), machine learning based on mapping of IgE epitope and motif search (13) or new approaches considering allergen-IgE interaction (14). It is highly likely that these advanced bioinformatic tools will provide higher sensitivity, specificity, accuracy, and improve allergenicity prediction. Furthermore, bioinformatic screening should also consider additional characteristics of proteins beyond its potential for cross-reactivity. These tools can also be used to provide information on the relatedness of a novel protein with commonly consumed proteins and the evolutionary distance between proteins relevant for allergenicity (15). However, advanced bioinformatic tools are not routinely used in the risk assessment process.

Exceptions exist and progressive bioinformatic tools have been developed for predicting the risk of proteins triggering celiac disease (16). The main elements which improved the bioinformatics tools used for celiac disease, for example, are: (i) a definition of clear inclusion criteria for database formation (17); (ii) a ranking strategy of immunodominant T-cell epitopes according to their clinical relevance and related features (18); and (iii) the development of a software tool for peptide binding prediction to HLA proteins (19). However, for allergenicity, current *in silico* approaches heavily rely on expert judgement to interpret *a posteriori* the outcome of the bioinformatic analysis. Because similarity search outcomes may change depending on the database used, (e.g., Allergenonline^1^, CompareDatabase^2^, Allergome^3^, WHO/IUIS^4^), it can lead to a lack of harmonisation, reproducibility, and transparency in the risk assessment process (8). It is imperative to refine databases so that they are fit-for-purpose for the allergenicity assessment (8, 20–24). To this end, the clinical relevance of known allergens in a given database should be defined *a priori* where allergens are ranked in terms of their clinical relevance, and are associated with specific risk assessment follow-up actions depending on the clinical relevance of the findings (20, 25). It will also be necessary to validate new bioinformatic tools using a comprehensive set of positive and negative control allergens.

### 2.2 *In vitro* tests

*In vitro* methods for the allergenicity assessment include protein stability measurements, e.g., classical pepsin resistance test and denaturation under differing pH and temperature conditions, and immunological assays, e.g., ELISAs and immunoblotting with human sera (Figure 1) (10, 26, 27). The most commonly used is the classical pepsin resistance test, which provides information on the stability of the proteins under acidic conditions and is useful in the weight-of-evidence approach. However, the test is poorly predictive of allergy, possibly because there is not a single intrinsic characteristic of proteins leading to allergenicity, and it does not mimic the physiologic conditions of gastric digestion (16, 28, 29). It is likely that understanding the influence of intestinal digestion on the fate of the proteins in the gastrointestinal tract and how they interact with relevant cells may improve predictability (29, 30), which could be achieved by improving the characterization of digestion products, e.g., molecular size, persistence, abundance, etc (16, 31). For instance, one new interesting approach is *in vitro* protein degradation studies, which simulate sequential gastric digestion followed by an intestinal digestion phase (32, 33). Because one of the most prominent traits attributed to known food allergens is protein stability (34–37), it will be crucial to optimize *in vitro* testing taking into account the following aspects: protein stability during heating and other processing procedures, pH changes and proteolysis, and physical stability, including aggregation. Consideration of industrial processing is critical and is emphasised in the guidelines on the effects of industrial processing of milk protein allergens, e.g., denaturation, the generation of new antigenic epitopes (38).

In GMO risk assessment, testing of the newly expressed proteins with human sera must be performed for the assessment if the source of the introduced gene is allergenic or if there is sequence homology similarity >35% with a known allergen (Figure 1) (10, 26, 27). However, it remains unclear i) how the testing should be specifically carried out; ii) why it is necessary to test human sera on all these cases; and iii) how additional elements such as the quality of the sequence homology similarity and the clinical relevance of the known allergen can be used to wave such requirements. Moreover, the difficulties identified in the assessment of newly expressed proteins become more complex when applied to whole foods.

There are an assortment of additional human cell and tissue models that might potentially be relevant for an allergenicity risk assessment such as biopsy-based models, coculture systems with epithelial and immune cells, precision cut organ slices, organoids, e.g., mini-gut cultures and organ-on-a chip models (gut-on-chip) (39–41). Moreover, there are *in vitro* models evaluating the potential sensitising capacity of food proteins such as antigen uptake via the intestinal mucosal barrier (42, 43). However, some of these models might need considerable work to ensure predictability and cost-effectiveness.

While *in vitro* assays are potentially invaluable, they require optimisation. For instance, test items and conditions will need to be standardized, information on interactions between proteins/fragments and the gastrointestinal tract/immune system need to be provided for the risk assessment process to ensure predictability.

### 2.3 *In vivo* studies

Mouse models of food allergy have been developed to understand further and elucidate underlying disease mechanisms (44). To date, it is not clear whether any of the models fully replicate human disease or whether they are able to predict protein allergenicity or adjuvanticity despite being used to assess the allergenicity and adjuvanticity risks of GMOs (45, 46). Nevertheless, where these models might be most useful is for further understanding the sensitizing potential of proteins, their cross-reactivity with other food proteins (46, 47), and the potential of novel proteins to act as adjuvants (46, 48–53). However, attention to experimental design, e.g., mouse strains, allergens, administration methods, and environmental factors, is crucial. Additionally, the model choice should be fit-for-purpose, multiple models might be needed, and combining data from *in silico, in vitro*, and *in vivo* models will likely improve predictability.

### 2.4 Other elements

Additional information from other sources may also improve the current risk assessment approach. For example, dietary exposure and eliciting dose data could be useful in the risk assessment process which are not clearly defined at the moment. Current regulatory guidelines focus on the hazard identification step of risk assessment. In future, we should explore possibilities to define more clearly what the role of exposure is in the overall risk assessment (8–10). Another possibility is the building of a framework with threshold levels of the most common and potent allergens, which could provide protection for people with food allergies (54). Indeed, a joint FAO/WHO expert Committee recently established recommended reference doses, based on the ED05 (max. 5% of the affected persons showing allergic reactions), for a series of major allergenic foods that meet the criterion of “exposure without appreciable health risk” (55). However, there are challenges that need be addressed, such as the lack of information for individual allergens and for food sources not considered common allergenic foods, as well as issues with inter-individual variability and with quality of clinical data. Nevertheless, it has been proposed that the use of information on the most common and potent allergens, as a worst-case scenario, should be able to cover other foods for which there is less available data (54).

Although pre-market monitoring has been successful, a post-market monitoring strategy could potentially prevent allergic reactions in subgroups of vulnerable individuals in the general population (56) and could address specific uncertainties arising from the pre-market assessment phase. However, it is crucial to consider the feasibility and practicality of including post-market monitoring requirements in the risk assessment process.

Specific risk assessment requirements might differ depending on the product under assessment and the regulatory frame under which it is evaluated. For example, the assessment of a simple protein or simple protein mixture vs. a complex protein mixtures or whole food leads to different challenges to the risk assessment process. Furthermore, the exposure scenario might differ depending on the product assessed. For instance, the assessment of a novel staple food is the most difficult allergenicity risk assessment scenario because staple foods are widely consumed and/or processed in different manners. Thus, a novel, widely consumed staple food is challenging to do a hazard, exposure or risk-based assessment.

## 3 Future needs for improving the allergenicity assessment

Continuous scientific advances over the last two decades have led to a functional asynchrony between the availability of safety standards and available scientific knowledge. As the numbers and complexity of new GMOs and new novel foods grow, there is a need for an overall revision of the allergenicity assessment objectives, still anchored on requirements and methodology established in late 90's. In 2022, EFSA published a scientific opinion on development needs for the allergenicity and protein safety assessment of food and feed products derived from biotechnology that provided short-and long-term recommendations. Therefore, it is necessary to revise and improve the allergenicity safety assessment. Here, we expand and prioritize advanced developmental stages, ready for implementation approaches to improve the current risk assessment including alternative/complementary methods to those already in place (e.g., *in silico* tools for cross-reactivity), and others that will need more development, research and consensus (e.g., *in vitro* tools for *de novo* sensitisation). Table 1 illustrates the priorities of these developments of which the top three are as follows:

(i) The development of a fit-for-purpose database, based on reliable and consensual inclusion criteria ensuring that only well-defined and characterised allergens are included. Ideally, the database should contain specific follow-up actions when similarities above thresholds with known allergens are identified depending on the clinical relevance and the quality of the similarity matches. Data curation and maintenance should also be specified;
(ii) The definition of a set of positive and negative control allergens together with the development of a specific validation testing process for *in silico*, *in vitro* and *in vivo* models. This process will need the development of a clear hypothesis relevant for allergenicity assessment and standardised experimental design ensuring appropriate statistical power under precise conditions and proper controls; and
(iii) Consensus on the purpose of an allergenicity risk assessment. A new frame of the purpose of the allergenicity assessment should be identified and internationally agreed where the role of exposure should be clarified, and consideration of the desired risk management outcome (e.g., preventing allergen sensitisation, accepting rare, potentially fatal reactions).

**Table 1:** Priorities of development needs for an improved allergenicity assessment.

Data collection	– Allergenic potency of certain allergenic foods and genetic differences of individuals – Component-resolved diagnostics in allergic patients – Prevalence and determinants of food allergy in animals (e.g. companion animals, farm) – Scaling and comparison of the allergenic potential for allergenic foods and individual allergens
	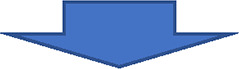
Build consensus	– Clinically relevant allergens with demonstrable potency in eliciting allergic reactions – Database for risk assessment purpose beyond classical yes/no classification – Reference set of proteins with varying allergenic potential for the development of improved predictive models for risk assessment—Allergens and no/weak allergens – Interaction between allergenic proteins with other components in food that influences their potency and stability and their potential as adjuvants – Reliable, accurate and sensitive methods to assess the potency, stability and potential allergenicity and adjuvant activity of allergens
	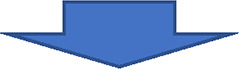
Develop new tools	– New *in silico*, *in vitro*, *ex vivo* and *in vivo* approaches able to predict allergenicity of food proteins – Validate and standardise methodology, experimental design, and read-outs – Adverse outcome pathway (AOP) can be applied to food sensitization/elicitation to support new allergenicity assessment strategies – Establish standardised test materials for the prediction of allergenicity, e.g., individual proteins and extracts (raw or processed), whole food matrix or a combination – Processing and preparation of test materials to cover any potential use for food/feed purposes or only over a product-based risk safety assessment – Characteristics of test materials related to protein stability, e.g. post-translational modifications, other biochemical and/or physicochemical properties – Data integration between experiments to allow for the extrapolation of broader conclusions than a single study – Standardise the experimental design to validate clinical context and integrate all data sets using multivariate models

New tools developed for allergenicity prediction should consider models for cross-reactivity (e.g., elicitation), sensitization and adjuvanticity, providing more precise information and clarity on how the weight-of-evidence approach is used, and the role of expert judgment in the overall safety assessment. Most importantly, any new tool/approach developed for its use in risk assessment should be proven to have better sensitivity, specificity and accuracy than current methods as well as being reproducible and cost-effective.

## Footnotes

^1^ http://www.allergenonline.org/

^2^ https://comparedatabase.org/

^3^ https://www.allergome.org/

^4^ https://allergen.org/
