# Supplementary material for: Research gaps and future needs for allergen prediction in food safety
Source: Front Allergy. 2024 Feb 19;5:1297547. doi: 10.3389/falgy.2024.1297547 (PMC10911423; doi:10.3389/falgy.2024.1297547)
Supplement: Supplementary file 1 [file Table1.docx]

**Supplementary information.** GMOs approved in the EU based on the International Service for the Acquisition of Agri-biotech Applications database [Accessed July 15, 2023]:

| Event Name and Code | Trade Name |
| --- | --- |
| Argentine Canola - *Brassica napus* : 14 Events |  |
| Name: GT73 (RT73)  Code: MON-ØØØ73-7 | Roundup Ready™ Canola |
| Name: HCN28 (T45)  Code: ACS-BNØØ8-2 | not available |
| Name: HCN92 (Topas 19/2)  Code: ACS-BNØØ7-1 | Liberty Link™ Innovator™ |
| Name: MON88302  Code: MON-883Ø2-9 | TruFlex™ Roundup Ready™ Canola |
| Name: MON88302 x MS8 x RF3  Code: MON-883Ø2-9 x ACS-BNØØ5-8 x ACS-BNØØ3-6 | InVigor™ x TruFlex™ Roundup Ready™ Canola |
| Name: MS1 (B91-4)  Code: ACS-BNØØ4-7 | InVigor™ Canola |
| Name: MS1 x RF1 (PGS1)  Code: ACS-BNØØ4-7 x ACS-BNØØ1-4 | InVigor™ Canola |
| Name: MS1 x RF2 (PGS2)  Code: ACS-BNØØ4-7 x ACS-BNØØ2-5 | InVigor™ Canola |
| Name: MS8  Code: ACS-BNØØ5-8 | InVigor™ Canola |
| Name: MS8 x RF3  Code: ACS-BNØØ5-8 x ACS-BNØØ3-6 | InVigor™ Canola |
| Name: MS8 x RF3 x GT73 (RT73)  Code: ACS-BNØØ5-8 x ACS-BNØØ3-6 x MON-ØØØ73-7 | not available |
| Name: RF1 (B93-101)  Code: ACS-BNØØ1-4 | InVigor™ Canola |
| Name: RF2 (B94-2)  Code: ACS-BNØØ2-5 | InVigor™ Canola |
| Name: RF3  Code: ACS-BNØØ3-6 | InVigor™ Canola |
| Carnation - *Dianthus caryophyllus* : 7 Events |  |
| Name: 11363 (1363A)  Code: FLO-11363-1 | Moonshadow™ |
| Name: 1226A (11226)  Code: FLO-11226-8 | Moonshade™ |
| Name: 123.2.38 (40644)  Code: FLO-4Ø644-4 | Moonlite™ |
| Name: 25958  Code: IFD-25958-3 | Moonberry™ |
| Name: 26407  Code: IFD-264Ø7-2 | Moonvelvet™ |
| Name: 959A (11959)  Code: FLO-11959-3 | Moonshade™ |
| Name: 988A (11988)  Code: FLO-11988-7 | Moonshade™ |
| Cotton - *Gossypium hirsutum L.* : 15 Events |  |
| Name: 281-24-236 x 3006-210-23 (MXB-13)  Code: DAS-24236-5 x DAS-21Ø23-5 | WideStrike™ Cotton |
| Name: 3006-210-23 x 281-24-236 x MON88913  Code: DAS-21Ø23-5 x DAS-24236-5 x MON-88913-8 | Widestrike™ Roundup Ready Flex™ Cotton |
| Name: GHB119  Code: BCS-GHØØ5-8 | not available |
| Name: GHB614  Code: BCS-GHØØ2-5 | GlyTol™ |
| Name: GHB614 x LLCotton25  Code: BCS-GHØØ2-5 x ACS-GHØØ1-3 | GlyTol™ Liberty Link™ |
| Name: GHB614 x T304-40 x GHB119  Code: BCS-GHØØ2-5 x BCS-GHØØ4-7 x BCS-GHØØ5-8 | Glytol™ x Twinlink™ |
| Name: GHB811  Code: BCS-GH811-4 | not available |
| Name: LLCotton25  Code: ACS-GHØØ1-3 | Fibermax™ Liberty Link™ |
| Name: MON1445  Code: MON-Ø1445-2 | Roundup Ready™ Cotton |
| Name: MON15985  Code: MON-15985-7 | Bollgard II™ Cotton |
| Name: MON15985 x MON1445  Code: MON-15985-7 x MON-Ø1445-2 | Roundup Ready™ Bollgard II™ Cotton |
| Name: MON531  Code: MON-ØØ531-6 | Bollgard™ Cotton, Ingard™ |
| Name: MON531 x MON1445  Code: MON-Ø531-6 x MON-Ø1445-2 | Roundup Ready™ Bollgard™ Cotton |
| Name: MON88913  Code: MON-88913-8 | Roundup Ready™ Flex™ Cotton |
| Name: T304-40  Code: BCS-GHØØ4-7 | not available |
| Maize - *Zea mays L.* : 55 Events |  |
| Name: 59122  Code: DAS-59122-7 | Herculex™ RW |
| Name: 59122 x MON88017  Code: DAS-59122-7 x MON-88Ø17-3 | not available |
| Name: 59122 x NK603  Code: DAS-59122-7 x MON-ØØ6Ø3-6 | Herculex™ RW Roundup Ready™ 2 |
| Name: Bt11 (X4334CBR, X4734CBR)  Code: SYN-BTØ11-1 | Agrisure™ CB/LL |
| Name: Bt11 x 59122 x MIR604 x TC1507 x GA21  Code: SYN-BTØ11-1 x DAS-59122-7 x SYN-IR6Ø4-5 x DAS-Ø15Ø7-1 x MON-ØØØ21-9 | Agrisure® 3122 |
| Name: Bt11 x GA21  Code: SYN-BTØ11-1 x MON-ØØØ21-9 | Agrisure™ GT/CB/LL |
| Name: Bt11 x MIR162  Code: SYN-BTØ11-1 x SYN-IR162-4 | Agrisure® Viptera™ 2100 |
| Name: Bt11 x MIR162 x GA21  Code: SYN-BTØ11-1 x SYN-IR162-4 x MON-ØØØ21-9 | Agrisure® Viptera™ 3110 |
| Name: BT11 x MIR162 x MIR604  Code: SYN-BTØ11-1 x SYN-IR162-4 x SYN-IR6Ø4-5 | Agrisure® Viptera™ 3100 |
| Name: Bt11 x MIR162 x MIR604 x GA21  Code: SYN-BTØ11-1 x SYN-IR162-4 x SYN-IR6Ø4-5 x MON-ØØØ21-9 | Agrisure® Viptera™ 3111, Agrisure® Viptera™ 4 |
| Name: Bt11 x MIR604  Code: SYN-BTØ11-1 x SYN-IR6Ø4-5 | Agrisure™ CB/LL/RW |
| Name: Bt11 x MIR604 x GA21  Code: SYN-BTØ11-1 x SYN-IR6Ø4-5 x MON-ØØØ21-9 | Agrisure™ 3000GT |
| Name: [Bt176 (176)](https://www.isaaa.org/gmapprovaldatabase/event/default.asp?EventID=127)  Code: SYN-EV176-9 | NaturGard KnockOut™, Maximizer™ |
| Name: DAS40278  Code: DAS-4Ø278-9 | Enlist™ Maize |
| Name: GA21  Code: MON-ØØØ21-9 | Roundup Ready™ Maize, Agrisure™GT |
| Name: GA21 x MON810  Code: MON-ØØØ21-9 x MON-ØØ81Ø-6 | Roundup Ready™ YieldGard™ maize |
| Name: MIR162  Code: SYN-IR162-4 | Agrisure™ Viptera |
| Name: MIR162 x GA21  Code: SYN-IR162-4 x MON-ØØØ21-9 | not available |
| Name: MIR162 x MIR604  Code: SYN-IR162-4 x SYN-IR6Ø4-5 | not available |
| Name: MIR162 x MIR604 x GA21  Code: SYN-IR162-4 x SYN-IR6Ø4-5 x MON-ØØØ21-9 | not available |
| Name: MIR604  Code: SYN-IR6Ø4-5 | Agrisure™ RW |
| Name: MIR604 x GA21  Code: SYN-IR6Ø4-5 x MON-ØØØ21-9 | Agrisure™ GT/RW |
| Name: MON810  Code: MON-ØØ81Ø-6 | YieldGard™, MaizeGard™ |
| Name: MON810 x MON88017  Code: MON-ØØ81Ø-6 x MON-88Ø17-3 | YieldGard™ VT Triple |
| Name: MON863  Code: MON-ØØ863-5 | YieldGard™ Rootworm RW, MaxGard™ |
| Name: MON863 x MON810  Code: MON-ØØ863-5 x MON-ØØ81Ø-6 | YieldGard™ Plus |
| Name: MON863 x MON810 x NK603  Code: MON-ØØ6Ø3-6 x MON-ØØ81Ø-6 x MON-ØØ863-5 | YieldGard™ Plus with RR |
| Name: MON863 x NK603  Code: MON-ØØ863-5 x MON-ØØ6Ø3-6 | YieldGard™ RW + RR |
| Name: MON87427 x MON89034 x NK603  Code: MON-87427-7 x MON-89Ø34-3 x MON-ØØ6Ø3-6 | not available |
| Name: MON87460  Code: MON-8746Ø-4 | Genuity® DroughtGard™ |
| Name: MON88017  Code: MON-88Ø17-3 | YieldGard™ VT™ Rootworm™ RR2 |
| Name: MON89034  Code: MON-89Ø34-3 | YieldGard™ VT Pro™ |
| Name: MON89034 x 59122  Code: MON-89Ø34-3 x DAS-59122-7 | not available |
| Name: MON89034 x 59122 x MON88017  Code: MON-89Ø34-3 x DAS-59122-7 x MON-88Ø17-3 | not available |
| Name: MON89034 x MON88017  Code: MON-89Ø34-3 x MON-88Ø17-3 | Genuity® VT Triple Pro™ |
| Name: MON89034 x NK603  Code: MON-89Ø34-3 x MON-ØØ6Ø3-6 | Genuity® VT Double Pro™ |
| Name: MON89034 x TC1507  Code: MON-89Ø34-3 x DAS-Ø15Ø7-1 | not available |
| Name: MON89034 x TC1507 x 59122  Code: MON-89Ø34-3 x DAS- Ø15Ø7-1 x DAS-59122-7 | not available |
| Name: MON89034 x TC1507 x MON88017  Code: MON-89Ø34-3 x DAS-Ø15Ø7-1 x MON-88Ø17-3 | not available |
| Name: MON89034 x TC1507 x MON88017 x 59122  Code: MON-89Ø34-3 x DAS-Ø15Ø7-1 x MON-88Ø17-3 x DAS-59122-7 | Genuity® SmartStax™ |
| Name: MON89034 x TC1507 x NK603  Code: MON-89Ø34-3 x DAS-Ø15Ø7-1 x MON-ØØ6Ø3-6 | Power Core™ |
| Name: MON89034 x TC1507 x NK603 x MIR162 x DAS40278  Code: MON-89Ø34-3 x DAS-Ø15Ø7-1 x MON-ØØ6Ø3-6 x SYN-IR162-4 x DAS-4Ø278-9 | Power Core™ x MIR162 x Enlist™ |
| Name: MZIR098  Code: SYN-ØØØ98-3 | not available |
| Name: NK603  Code: MON-ØØ6Ø3-6 | Roundup Ready™ 2 Maize |
| Name: NK603 x MON810  Code: MON-ØØ6Ø3-6 x MON-ØØ81Ø-6 | YieldGard™ CB + RR |
| Name: NK603 x T25  Code: MON-ØØ6Ø3-6 x ACS-ZMØØ3-2 | Roundup Ready™ Liberty Link™ Maize |
| Name: T25  Code: ACS-ZMØØ3-2 | Liberty Link™ Maize |
| Name: TC1507  Code: DAS-Ø15Ø7-1 | Herculex™ I, Herculex™ CB |
| Name: TC1507 × 59122 × MON810 × MIR604 x NK603  Code: DAS-Ø15Ø7-1 × DAS-59122-7 × MON-ØØ81Ø-6 × SYN-IR6Ø4-5 x MON-ØØ6Ø3-6 | Optimum™ Intrasect Xtreme |
| Name: TC1507 x 59122  Code: DAS-Ø15Ø7-1 x DAS-59122-7 | Herculex XTRA™ |
| Name: TC1507 x 59122 x MON88017  Code: DAS-Ø15Ø7-1 x DAS-59122-7 x MON-88Ø17-3 | not available |
| Name: TC1507 x 59122 x NK603  Code: DAS-Ø15Ø7-1 x DAS-59122-7 x MON-ØØ6Ø3-6 | Herculex XTRA™ RR |
| Name: TC1507 x MON810 x MIR162 x NK603  Code: DAS-Ø15Ø7-1 x MON-ØØ81Ø-6 x SYN-IR162-4 x MON-ØØ6Ø3-6 | not available |
| Name: TC1507 x MON88017  Code: DAS-Ø15Ø7-1 x MON-88Ø17-3 | not available |
| Name: TC1507 x NK603  Code: DAS-Ø15Ø7-1 x MON-ØØ6Ø3-6 | Herculex™ I RR |
| Potato - *Solanum tuberosum L.* : 1 Event |  |
| Name: EH92-527-1  Code: BPS-25271-9 | Amflora™ |
| Soybean - *Glycine max L.* : 22 Events |  |
| Name: A2704-12  Code: ACS-GMØØ5-3 | Liberty Link® soybean |
| Name: A5547-127  Code: ACS-GMØØ6-4 | Liberty Link® soybean |
| Name: CV127  Code: BPS-CV127-9 | Cultivance |
| Name: [DAS44406-6](https://www.isaaa.org/gmapprovaldatabase/event/default.asp?EventID=345)  Code: DAS-444Ø6-6 | not available |
| Name: DAS68416-4  Code: DAS-68416-4 | not available |
| Name: DAS81419  Code: DAS-81419-2 | not available |
| Name: DAS81419 x DAS44406  Code: DAS-81419-2 x DAS-444Ø6-6 | Conkesta Enlist E3™ Soybean |
| Name: DP305423  Code: DP-3Ø5423-1 | Treus™, Plenish™ |
| Name: DP305423 x GTS 40-3-2  Code: DP-3Ø5423-1 x MON-Ø4Ø32-6 | not available |
| Name: DP356043  Code: DP-356Ø43-5 | Optimum GAT™ |
| Name: FG72 (FGØ72-2, FGØ72-3)  Code: MST-FGØ72-2 | not available |
| Name: FG72 x A5547-127  Code: MST-FGØ72-2 x ACS-GMØØ6-4 | Liberty Link® GT27™ |
| Name: GTS 40-3-2 (40-3-2)  Code: MON-Ø4Ø32-6 | Roundup Ready™ soybean |
| Name: MON87701  Code: MON-877Ø1-2 | not available |
| Name: MON87701 x MON89788  Code: MON-877Ø1-2 x MON-89788-1 | Intacta™ Roundup Ready™ 2 Pro |
| Name: MON87705  Code: MON-877Ø5-6 | Vistive Gold™ |
| Name: MON87705 x MON89788  Code: MON-877Ø5-6 x MON-89788-1 | not available |
| Name: MON87708  Code: MON-877Ø8-9 | Genuity® Roundup Ready™ 2 Xtend™ |
| Name: MON87708 x MON89788  Code: MON-877Ø8-9 x MON-89788-1 | not available |
| Name: MON87708 x MON89788 x A5547-127  Code: MON-877Ø8-9 x MON-89788-1 x ACS-GMØØ6-4 | not available |
| Name: MON87769  Code: MON87769-7 | not available |
| Name: MON89788  Code: MON-89788-1 | Genuity® Roundup Ready 2 Yield™ |
| Sugar Beet - *Beta vulgaris* : 1 Event |  |
| Name: H7-1  Code: KM-ØØØH71-4 | Roundup Ready™ sugar beet |
| Tobacco - *Nicotiana tabacum L.* : 1 Event |  |
| Name: C/F/93/08-02  Code: not available | not available |
